# Accumulation of cholesterol, triglycerides and ceramides in hepatocellular carcinomas of diethylnitrosamine injected mice

**DOI:** 10.1186/s12944-021-01567-w

**Published:** 2021-10-10

**Authors:** Elisabeth M. Haberl, Rebekka Pohl, Lisa Rein-Fischboeck, Marcus Höring, Sabrina Krautbauer, Gerhard Liebisch, Christa Buechler

**Affiliations:** 1 Department of Internal Medicine I, Regensburg University Hospital, 93053 Regensburg, Germany; 2 Institute of Clinical Chemistry and Laboratory Medicine, Regensburg University Hospital, 93053 Regensburg, Germany

**Keywords:** *De novo* lipogenesis, PGC1alpha, Phospholipids, p53

## Abstract

**Background:**

Dysregulated lipid metabolism is critically involved in the development of hepatocellular carcinoma (HCC). The respective metabolic pathways affected in HCC can be identified using suitable experimental models. Mice injected with diethylnitrosamine (DEN) and fed a normal chow develop HCC. For the analysis of the pathophysiology of HCC in this model a comprehensive lipidomic analysis was performed.

**Methods:**

Lipids were measured in tumor and non-tumorous tissues by direct flow injection analysis. Proteins with a role in lipid metabolism were analysed by immunoblot. Mann-Whitney U-test or paired Student´s t-test were used for data analysis.

**Results:**

Intra-tumor lipid deposition is a characteristic of HCCs, and di- and triglycerides accumulated in the tumor tissues of the mice. Peroxisome proliferator-activated receptor gamma coactivator 1 alpha, lipoprotein lipase and hepatic lipase protein were low in the tumors whereas proteins involved in *de novo* lipogenesis were not changed. Higher rates of *de novo* lipogenesis cause a shift towards saturated acyl chains, which did not occur in the murine HCC model. Besides, LDL-receptor protein and cholesteryl ester levels were higher in the murine HCC tissues. Ceramides are cytotoxic lipids and are low in human HCCs. Notably, ceramide levels increased in the murine tumors, and the simultaneous decline of sphingomyelins suggests that sphingomyelinases were involved herein. DEN is well described to induce the tumor suppressor protein p53 in the liver, and p53 was additionally upregulated in the tumors.

**Conclusions:**

Ceramides mediate the anti-cancer effects of different chemotherapeutic drugs and restoration of ceramide levels was effective against HCC. High ceramide levels in the tumors makes the DEN injected mice an unsuitable model to study therapies targeting ceramide metabolism. This model is useful for investigating how tumors evade the cytotoxic effects of ceramides.

**Supplementary Information:**

The online version contains supplementary material available at 10.1186/s12944-021-01567-w.

## Introduction

Hepatocellular carcinoma is a hard-to-cure malignancy and the incidence has progressively increased over the last decades [1–3]. Metabolic reprogramming is a hallmark of cancers during disease initiation and progression. Various studies have documented a higher expression of enzymes involved in *de novo* lipogenesis in human HCC tissues [4–8]. Ablation of fatty acid synthase (FAS) prevented the proliferation of HCC cells *in-vitro* and also delayed hepatocarcinogenesis in experimental models [9–12]. Further analysis showed that cholesterol biosynthesis was enhanced upon blockage of FAS in HCC cell lines and in the murine liver [10]. Of note, ablation of fatty acid and cholesterol biosynthesis completely prevented tumorigenesis in the liver of a murine HCC model induced by loss of Phosphatase and Tensin homolog and overexpression of c-Met [10]. Peroxisome proliferator-activated receptor gamma coactivator 1 alpha (PGC1alpha) increases fatty acid oxidation and lowers hepatic triglyceride storage and secretion [13]. PGC1alpha was downregulated in human HCC tissues and low expression was associated with a poor prognosis. Thus, emerging evidence indicates that lipids are important for the development and progression of HCC and may be targets for HCC therapies [5, 7, 10].

Pathogenesis of HCC is highly complex, and persistent inflammation is well known to promote carcinogenesis [14–16]. Strikingly, increasing evidence points at a functional connection of lipid metabolism and inflammation [15]. Saturated fatty acids are well described to activate pro-inflammatory signalling pathways in immune cells [17]. Liver steatosis is associated with an increased synthesis of inflammatory proteins by hepatocytes and leads to the activation of Kupffer cells [18]. Dietary fat induces the production of inflammatory cytokines in adipose tissues that may play a role in hepatic inflammation [19, 20]. PGC1alpha downregulation is associated with lipid deposition, oxidative stress and inflammation [21]. Liver steatosis was furthermore associated with an altered gut microbiota, higher intestinal permeability and increased gut-derived endotoxin levels, again linking lipid metabolism and inflammation [22].

The cancer lipidome of HCC patients has been analysed in a few studies so far [5]. These analyses described that triglycerides accumulated in human HCC tissues [23–25]. Aberrant activation of *de novo* lipogenesis favours the accumulation of saturated fatty acids and there was a shift from polyunsaturated to saturated lipids in human HCCs [24–26].

Deposition of excess cholesterol was also noted in human HCC tissues [27, 28]. Cholesterol biosynthesis and uptake is regulated by sterol regulatory element binding protein (SREBP) 2 [29]. SREBP2 increases the expression of various genes such as 3-hydroxy-3-methyl-glutaryl-CoA reductase and the low-density lipoprotein receptor (LDL-R). Emerging evidence indicates that blockage of SREBP2 may be effective to treat different cancers such as HCC [30]. Hepatic uptake of LDL is also regulated by proprotein convertase subtilisin/kexin type 9 (PCSK9), which induces the lysosomal degradation of the LDL-R [31]. Low expression of PCSK9 and high expression of the LDL-R in human HCCs [32] suggests that this pathway contributes to cholesterol overload of the tumors.

Ceramides are a relatively well studied lipid class and have a role in various cellular processes [33]. There is convincing evidence that ceramide function depends on its acyl-chain length [5, 34]. The long-chain species (C16 - C20) increase insulin resistance, cell death and oxidative stress and the very long-chain derivatives (C22 - C24) have the opposite effects [5, 34]. Ceramide levels were low in human HCCs, and thus, it was supposed that shorter chain ceramides protect from tumor progression [24–26, 35]. Short-chain ceramides delivered by nanoliposomes were indeed effective against HCC [33, 36–38]. Notably, chemotherapeutics induce ceramide production that mediates the anti-proliferative and pro-apoptotic effects of these drugs [33].

Potential HCC therapies are being tested in suitable experimental models [39]. The most widely used approach to induce liver cancer in rodents is a single injection of diethylnitrosamine (DEN) to young mice. The time required for tumor development varies with DEN dose, mouse strain, and sex [40].

Only little data is available on the lipidome of murine tumors in the DEN model. Considering that lipids have a central role in cell viability, proliferation and inflammation [5, 18, 41, 42], a detailed lipidomic analysis will help to further understand tumor pathology and to develop lipid-based therapies. Moreover, a comprehensive characterization of the HCC lipidome makes it possible to choose the most appropriate murine model.

Mice fed a low methionine, choline-deficient chow, when injected with DEN at a young age, develop HCC. Unexpectedly, expression of FAS and acetyl-CoA-carboxylase was low in the tumors. Accordingly, triglycerides and diglycerides were reduced in the HCC tissues of these animals compared to non-tumor liver tissues [43]. A prominent decline of ceramides was not observed in the tumors of those mice. Thus, the tumor lipidome of these animals did not resemble the lipid changes observed in human HCCs [5, 43].

Male C3H/HeNRj (25 mg/kg DEN injected at 18–21 days of age), which were fed a normal chow, accumulated diglycerides, triglycerides and cholesterol in the tumors [44]. Excess of these lipid classes was described in human HCCs [5]. Aim of the present investigation was to clarify whether these mice are a suitable model to study the role of lipid dysregulation described in human HCC. For this purpose, a comprehensive analysis of the tumor lipidome was performed. Besides, several proteins with a role in cholesterol and triglyceride metabolism were analysed to identify the proteins underlying these changes in lipid composition.

## Materials and methods

### Animals

The animals used in the present study served as controls in a previous investigation and were injected with adeno-associated virus 8 (AAV8) particles without any cloned DNA [44]. AAV8 vectors are being used in various animal models without any severe adverse effects [45]. Thus it is unlikely that AAV8 greatly affects the lipidome [46].

Animal model: Male C3H/HeNRj mice (Janvier Labs, Le Genest-Saint-Isle, France) were injected with DEN (Sigma, Taufkirchen, Germany; 25 µg/g body weight) at 18–21 days of age. A total of 24 weeks later, AAV8 without any cloned DNA (10^12^ particles per mouse) were intraperitoneally injected, and the mice were killed 13 weeks later. These mice were fed a standard chow (V1124-300, Mouse breading 10 mM autoclavable, Ssniff, Soest, Germany) throughout the study. Normal liver tissues of 12 mice and tumorous liver tissues of 10 mice were used in the current analysis. Mice killed 37 weeks after DEN injection developed a variety of liver tumors. Tumor diameter ranged from < 1 mm to > 10 mm [44]. Liver tumors that were clearly distinguishable from normal liver tissue were excised using a pair of binoculars. These tissues are suitable for the purpose of the current investigation where normal liver and tumors were compared. Reuse of these tissues also considers the 3Rs to improve animal welfare [47].

Mice had free access to water and food and were housed in a 21 ± 1 °C controlled room under a 12 h light-dark cycle. All procedures were in accordance with the institutional and governmental regulations for animal use (Approval number 54-2532.1-21/14, 03,11,2014).

### Mass spectrometric analysis

Lipids were extracted from 2 mg liver tissues as was described by Bligh and Dyer [48]. Non-naturally occurring lipid species, which served as internal standards, were added during lipid extraction (Internal standards: PC 14:0/14:0, PC 22:0/22:0, PE 14:0/14:0, PE 20:0/20:0 (di-phytanoyl), PS 14:0/14:0, PS 20:0/20:0 (di-phytanoyl), PI 17:0/17:0, LPC 13:0, LPC 19:0, LPE 13:0, Cer d18:1/14:0, Cer d18:1/17:0, D7-FC, CE 17:0, CE 22:0, TG 51:0, TG 57:0, DG 28:0 and DG 40:0). The chloroform phase was vacuum dried and the leftover was solubilized in chloroform/methanol/2-propanol (1:2:4 v/v/v) with 7.5 mM ammonium formate (for high resolution mass spectrometry) or in methanol/chloroform (3:1, v/v) with 7.5 mM ammonium acetate (for low mass resolution tandem mass spectrometry).

Lipids were analyzed by direct flow injection analysis and this was described elsewhere [43, 49–52]. Direct flow injection analysis (FIA) using a triple quadrupole mass spectrometer (FIA-MS/MS; QQQ triple quadrupole) and a hybrid quadrupole-Orbitrap mass spectrometer (FIA-FTMS; high mass resolution) were used. FIA-MS/MS (QQQ) was carried out in positive ion mode as was already described [49, 52]. A fragment ion of *m/z* 184 was employed for lysophosphatidylcholines (LPCs) [50]. The neutral loss applied for phosphatidylethanolamine (PE) was 141, for phosphatidylserine (PS) was 185, and for phosphatidylinositol (PI) was 277 [53]. Sphingosine based ceramides (Cer) were determined using a fragment ion of *m/z* 264 [51].

The Fourier Transform Mass Spectrometry (FIA-FTMS) method was described by Höring et al. [54]. Triglycerides (TGs), diglycerides (DGs) and cholesteryl ester (CE) were determined in positive ion mode FTMS in range *m/z* 500–1000 for 1 min with a maximum injection time (IT) of 200 ms, an automated gain control of 1*10^6^, three microscans and a target resolution of 140,000 (at *m/z* 200). Phosphatidylcholine (PC) and sphingomyelin (SM) were measured in range *m/z* 520–960. Multiplexed acquisition was used for the [M + NH_4_]^+^ of free cholesterol (FC) (*m/z* 404.39) and D_7_-cholesterol (*m/z* 411.43) for 0.5 min acquisition time, with a normalized collision energy of 10 %, an IT of 100 ms, automated gain control of 1*10^5^, isolation window of 1 Da, and a target resolution of 140,000 (at m/z 200). Data processing by the use of the ALEX software [55] and self-programmed Macros (Microsoft Excel 2010) was described previously [56]. Lipid species were noted according to the shorthand notation of lipid structures derived from mass spectrometry analysis [57]. For QQQ glycerophospholipid species even numbered carbon chains were assumed. Liver lipids are given as nmol/mg wet weight.

### Immunoblotting

Immunoblotting was carried out as described [58]. Antibodies for glyceraldehyde-3-phosphate dehydrogenase (GAPDH) (order number: 2118), phosphorylated (p)ACC (order number: 3661), pAMP-activated protein kinase (AMPK; order number: 2351), AMPK (order number: 2532), hormone sensitive lipase (HSL) (order number: 4107), stearoyl-CoA-reductase (SCD1) (order number: 2794), cyclophilin A (order number: 2175) and fatty acid synthase (FAS) (order number: 3189) were from Cell Signaling (Frankfurt am Main, Germany). Hepatic lipase (LIPC) antibody (order number: LS-C331464-50) was from LSBio (Seattle, Washington, United States). Apolipoprotein (Apo) B (order number: MA5-35458 ), ApoE (order number: ab947), ApoAII (order number: 178424), lipoprotein lipase (LPL) (order number: BS-23,362), diacylglycerol-O-acyltransferase (DGAT) 2 (order number: PA5-103785), SREBP1c (order number: MS-1207-P1ABX) and manganese superoxide dismutase (MnSOD) (order number: LF-PA0021) antibodies were from Thermo Fisher Scientific (Schwerte, Germany). Acetyl-CoA-carboxylase (ACC) (order number: MAB 6898), CD36 (order number: NB400-145), scavenger receptor BI (order number: NB400-104) and DGAT1 (order number: NB-110-4148755) antibodies were from Novus Biologicals (Wiesbaden-Nordenstadt, Germany). The PCSK9 (order number: AF3985), LDL-receptor (order number: AF2255-SP) and p53 (order number: AF1355) antibodies were from R&D Systems (Wiesbaden-Nordenstadt, Germany). PGC1alpha antibody was from Abcam (Cambridge, UK, order number: ab106814). SREBP2 antibody was from Caymen Chemical (Hamburg. Germany; order number: 10,007,663).

### Real-time RT-PCR

RNA was purified using the AllPrep DNA/RNA/Protein Mini Kit (Qiagen, Hildesheim, Germany). RT-PCR was performed as described in detail [44]. LDL-receptor was amplified with 5´ GAT GGC TAT ACC TAC CCC TCA A 3´ and 5´ CCT TTT CTG TCC CCA GAC AA 3´. PGC1alpha was amplified with 5´ GGA ATG CAC CGT AAA TCT GC 3´ and 5´ AAA ATC CAG AGA GTC ATA CTT GCT C 3´. For normalisation, cyclophilin A was used and primers were 5´ AAC ACA AAC GGT TCC CAG TT 3´and 5´ TTG AAG GGG AAT GAG GAA AA 3´.

### GeneChip analysis

The Mouse Gene 2.1. ST Array (Affymetrix, Schwerte, Germany) was hybridized with RNA from normal liver and HCC tissues of five animals. Hybridization and data analysis was performed by the Kompetenzzentrum für Fluoreszente Bioanalytik (Regensburg, Germany). The *P*-value for AFP regulation (Table 2), which was shown previously to be induced in tumors of DEN-injected mice [59], was chosen as cut off value.

### Statistical analysis

Data are shown as box plots. Outliers are identified by small circles and extreme values are marked with stars. Orange and red circles in the figures are the individual values measured. Quantification of proteins was done using ImageJ [60]. Data of proteins, which were not changed in the tumor tissues, are shown as mean ± standard deviation. Statistical differences were calculated by Mann-Whitney U-test or paired Students´t-test. Spearman correlation analysis was also used (SPSS Statistics 25.0 program; IBM, Leibniz Rechenzentrum, Munich, Germany). Values of *P* < 0.05 were considered as significant.

## Results

### Triglyceride and diglyceride accumulation in the tumor tissues

**Fig. 1:**
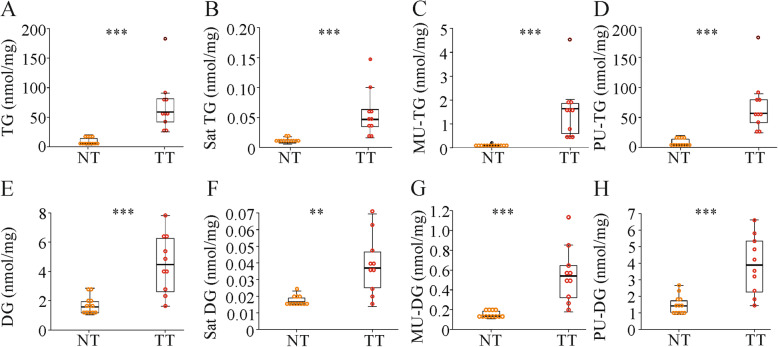
Levels of triglycerides (TGs) and diglycerides (DGs) in the normal tissues (NT) and tumor tissues (TT) of mice injected with diethylnitrosamine (*n* = 9–12 mice). **a** Hepatic TGs. **b** Saturated (sat) TGs. **c** Monounsaturated (MU) TGs. **d** Polyunsaturated (PU) TGs. **e** Hepatic DGs. **f** Sat DGs. **g** MU-DGs. **h** PU-DGs. ** *P* < 0.01; *** *P* < 0.001

Triglyceride (TG) deposition was observed in the murine tumors (Fig. 1a and [44]). Saturated, monounsaturated (MU) and polyunsaturated (PU) TGs were increased (Fig. 1b-d). Diglycerides (DGs) also accumulated in the cancer tissues (Fig. 1e and [44]), and saturated, MU-DG and PU-DG levels were high in the tumors (Fig. 1f-h). The PU/saturated TG ratio was 545 (271–2001) in the normal liver and increased to 1246 (750–1987) in the tumors (*P* = 0.017). The PU/saturated DG ratio was 78 (45–133) in the non-tumor tissues and 92 (76–194) in the HCC tissues (*P* = 0.069).

### Lipoprotein lipase, hepatic lipase and PGC1alpha are downregulated in the tumors

**Fig. 2:**
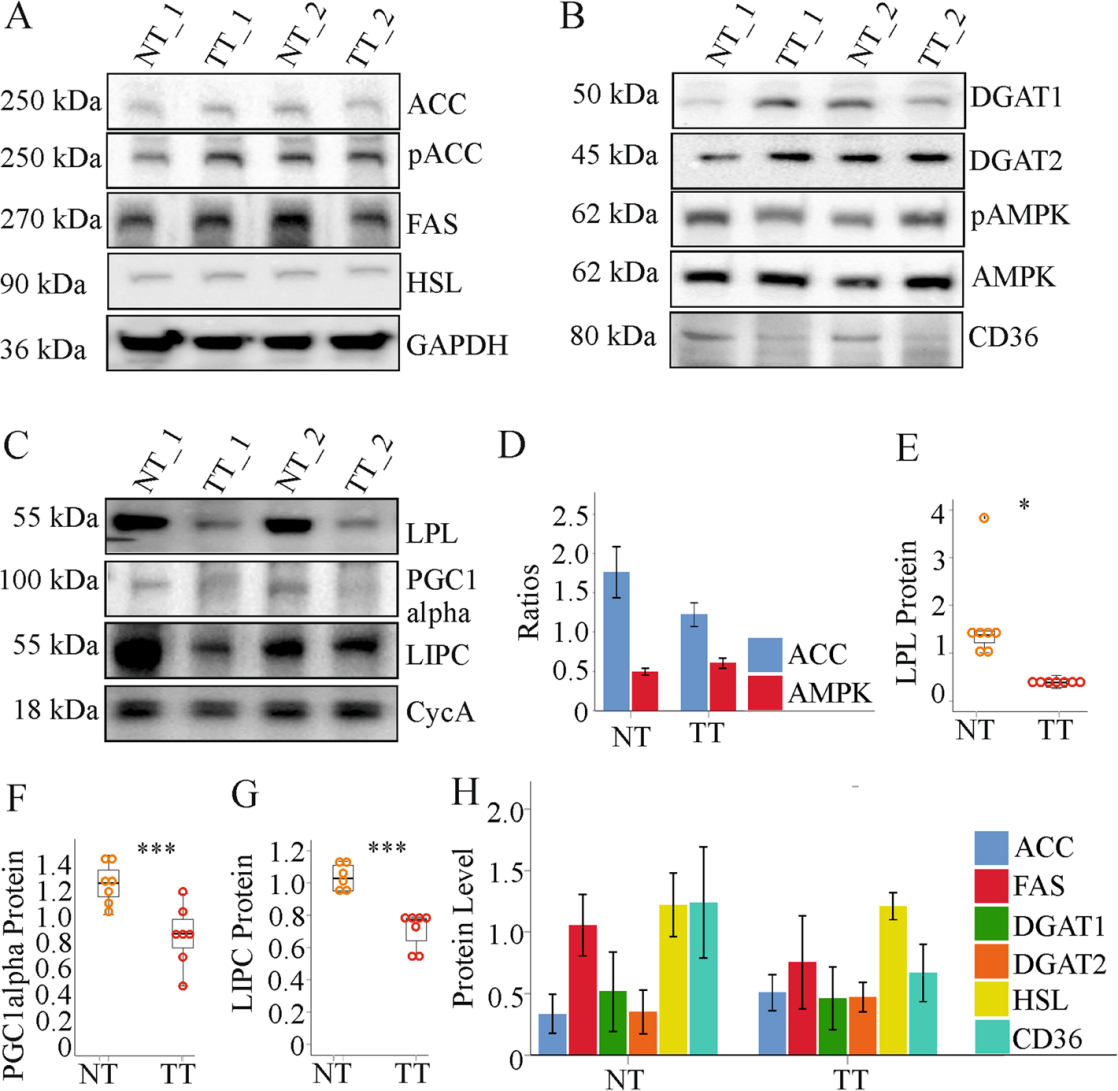
Enzymes with a role in TG synthesis in the normal tissues (NT) and tumor tissues (TT) of mice injected with diethylnitrosamine. **a** Expression of ACC, pACC, FAS, and HSL. **b** Expression of DGAT1, DGAT2, pAMPK, AMPK and CD36. **c** Expression of LPL, PGC1alpha and LIPC. Cyclophilin A (CycA) was a further housekeeping protein analysed. **d** Ratio of pACC/ACC and pAMPK/AMPK in NT and TT. **e** Quantification of LPL protein. **f** Quantification of PGC1alpha protein. **g** Quantification of LIPC protein. **h** Quantification of proteins not changed in the TT. (*n* = 6–7). * *P* < 0.05; *** *P* < 0.001

Activation of SREBP1c was comparable in non-tumor and tumor tissues of these mice (Supplementary Fig. 1a and [44]). Accordingly, stearoyl-CoA-reductase, fatty acid synthase (FAS) and acetyl-CoA carboxylase (ACC) levels were not induced in the tumors (Supplementary Fig. 1a and Fig. 2a, h). The ratio of phosphorylated to not phosphorylated ACC did not change in the tumors (Fig. 1a, d). Hormone sensitive lipase, diacylglycerol O-acyltransferase (DGAT) 1 and 2 were similar in tumor and non-tumor tissues (Fig. 2a, b, h). AMPK and its phosphorylated form were not changed in the tumors (Fig. 2b, d). CD36 tended to decline (*P* = 0.05), while lipoprotein lipase (LPL), PGC1alpha and hepatic lipase (LIPC) proteins were all low in the tumors (Fig. 2b, c, e - g). PGC1alpha mRNA expression was, however, not changed in the HCC tissues (Table 1).

**Table 1:** Analysis of gene expression by real-time PCR in normal tissue (NT) and tumor tissue (TT) of 9 mice per group. Cyclophilin A mRNA was used for normalisation (PGC1alpha, Peroxisome proliferator-activated receptor-gamma coactivator 1alpha; LDL, Low density lipoprotein)

Tissue	PGC1alpha	LDL-receptor
NT	1.14 (0.64–2.09)	1.61 (1.02–2.63)
TT	1.25 (0.93–2.14)	1.82 (1.31–3.25)

Global gene expression analysis showed that carnitine palmitoyltransferase 2 (CPT2) mRNA was reduced in the tumors (Table 2). Further genes with a role in fatty acid oxidation such as CPT1a, acyl-Coenzyme A dehydrogenases, enoyl-Coenzyme A, hydroxysteroid (17-beta) dehydrogenase 4 and hydroxyacyl-Coenzyme A dehydrogenase/3-ketoacyl-Coenzyme A thiolase were not differentially expressed between normal and tumor tissues (Table 2 and data not shown). Of note, LIPC mRNA levels were reduced in the murine HCC tissues (Table 2).

**Table 2:** Gene expression from microarray experiments of CPT2, acyl-Coenzyme A dehydrogenase, medium chain (Acadm), hydroxyacyl-Coenzyme A dehydrogenase/3-ketoacyl-Coenzyme A thiolase/enoyl-Coenzyme A hydratase (trifunctional protein), alpha subunit (Hadha), LIPC and alpha-fetoprotein (AFP) in tumor and non-tumor tissues of 5 mice. The respective *P*-values are listed and the *P*-value for AFP was used as cut-off

Tissue	CPT2	Acadm	Hadha	LIPC	AFP
NT	6.9 (6.5–7.7)	35 (33–39)	26 (26–26)	8.2 (7.8–9.5)	2.0 (1.3–3.4)
TT	4.8 (4.6–6.1)	32 (31–34)	26 (26–29)	4.8 (3.6–5.9)	18.4 (14.1– 67.8)
*P*	0.00048	0.088	0.148	0.00125	0.00159

### Cholesterol, LDL-receptor and ApoB are induced in the tumor tissues

**Fig. 3:**
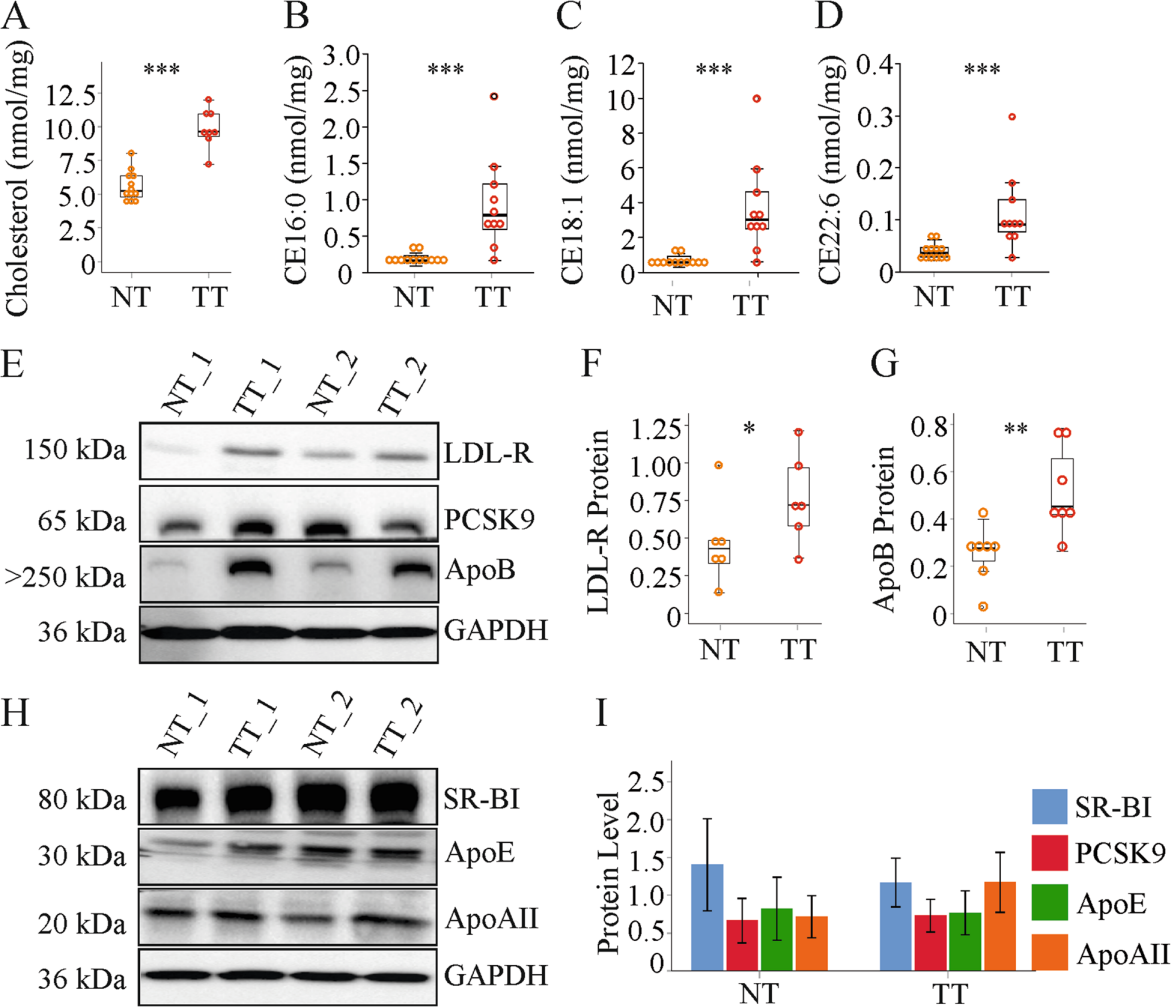
Levels of cholesterol and expression of proteins with a role in cholesterol metabolism in the normal tissues (NT) and tumor tissues (TT) of mice injected with diethylnitrosamine. **a** Hepatic cholesterol. **b** Cholesteryl ester (CE) 16:0 **c** CE18:1. **d** CE22:6. **e** Expression of the LDL-receptor (LDL-R), PCSK9 and ApoB protein. **f** Quantification of LDL-R protein. **g** Quantification of ApoB protein. **h** Expression of SR-BI, ApoE and ApoAII **i** Quantification of proteins not changed in the tumor tissues (*n* = 6–7). * *P* < 0.05, ** *P* < 0.01, *** *P* < 0.001

Total cholesterol levels were higher in the murine tumor tissues (Fig. 3a and [44]). Of the six analyzed cholesteryl ester (CE) species (CE16:0, 16:1, 18:1, 18:2, 20:4, 22:6) all but CE20:4 were significantly induced in the cancer tissues (Fig. 3b - d and Supplementatry Fig. 1b). Free cholesterol concentrations did not change (Supplementary Fig. 1b).

SREBP2 was, however, not overactivated in the tumors of the mice studied herein (Supplementatry Fig. 1c and [44]). Consequently, LDL-receptor mRNA was not changed in the cancer tissues (Table 1). LDL-receptor protein was nevertheless higher in the HCC tissues (Fig. 3e, f). Notably, apoB protein was strongly increased in the tumors (Fig. 3e, g).

Scavenger receptor BI, proprotein convertase subtilisin*/*kexin type 9 (PCSK9), apoE and apoAII were similar in tumor and non-tumor tissues (Fig. 2e, h, i).

### Ceramide species are elevated in the HCC tissues

**Fig. 4:**
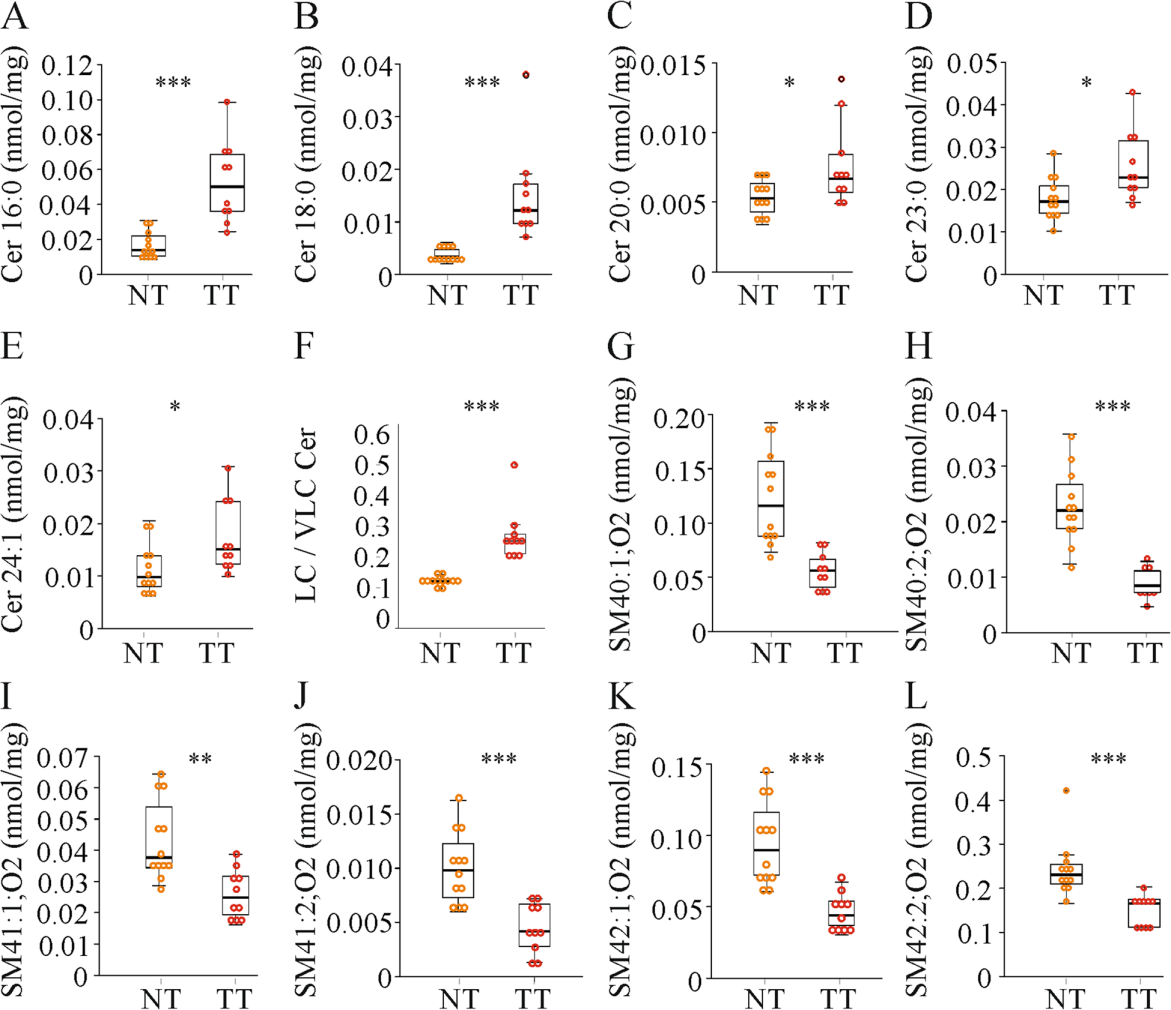
Hepatic ceramide (Cer) and sphingomyelin (SM) levels in the normal tissues (NT) and tumor tissues (TT) of mice injected with diethylnitrosamine (*n* = 8–12 mice). **a** Cer (d18:1/16:0). **b** Cer (d18:1/18:0). **c** Cer (d18:1/20:0). **d** Cer (d18:1/23:0). **e** Cer (d18:1/24:1). **f** Long-chain (LC; 16–20 N-acyl carbons) / very long-chain (VLC; >20 N-acyl carbons) Cer ratio. **g** SM40:1;O2. **h** SM40:2;O2. **i** SM41:1;O2. **j** SM41:2;O2 **k** SM42:1;O2. **l** SM42:2;O2 species. * *P* < 0.05, ** *P* < 0.01, *** *P* < 0.001

Ceramide levels were induced in the tumor tissues and of the seven different ceramide species measured, five were higher in the HCCs (Fig. 4a - e). The ratio of long-chain to very long-chain ceramide species was increased in the tumor tissues (Fig. 4f).

A decline of sphingomyelin (SM) levels was noticed in the HCC tissues (Fig. 4 g - l). Six of the nine different SM species measured were decreased in the HCC tissues (Fig. 4 g - l). The SM/ceramide ratio was markedly reduced in the tumors (data not shown).

### Phospholipids are hardly changed in the tumors

**Fig. 5:**
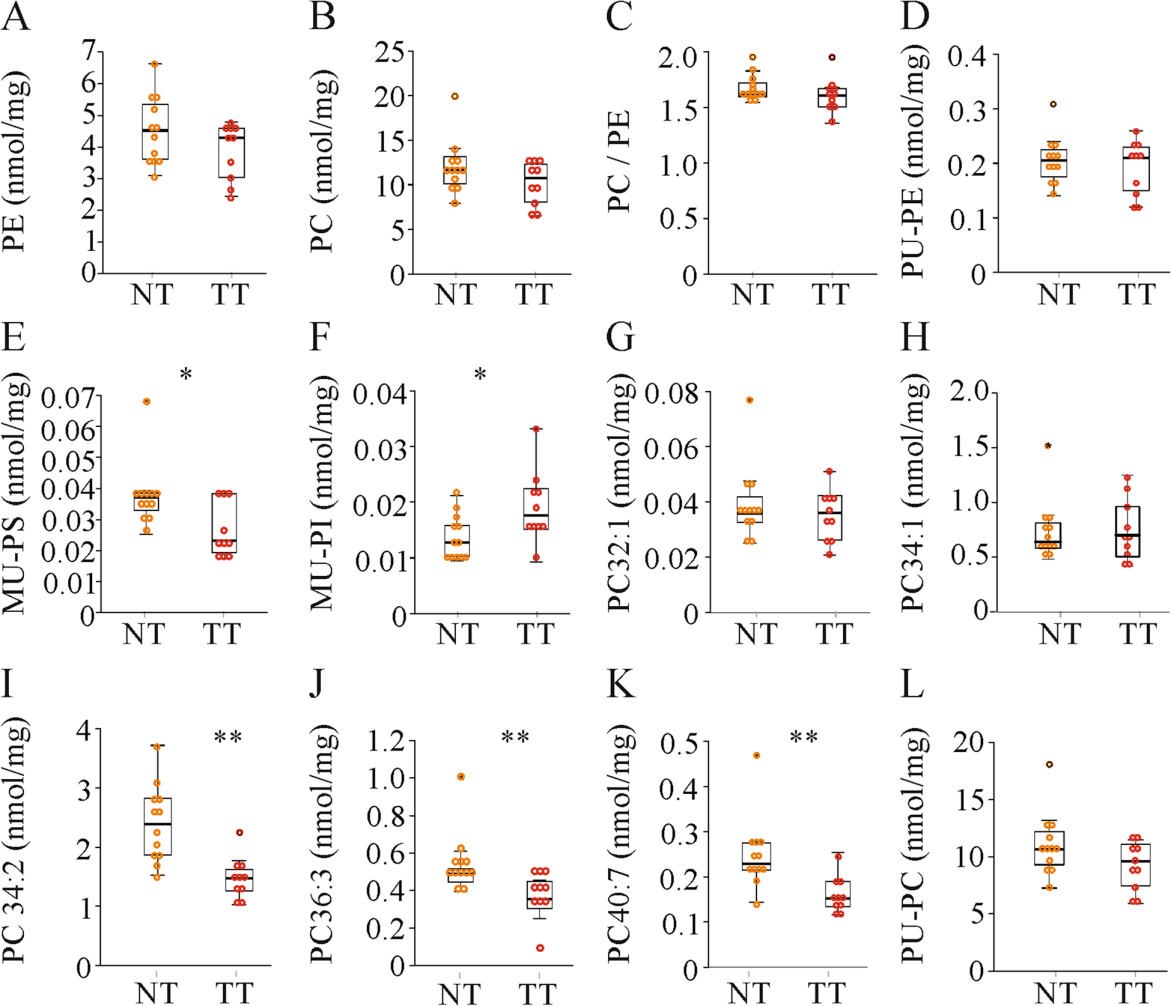
Hepatic phosphatidylcholine (PC), phosphatidylethanolamine (PE), phosphatidylserine (PS) and phosphatidylinositol (PI) levels in the normal tissues (NT) and tumor tissues (TT) of mice injected with diethylnitrosamine. (*n* = 10–12 mice). **a** PE. **b** PC. **c** PC / PE ratio. **d** Polyunsaturated (PU) PE. **e** Monounsaturated (MU) PS. **f** MU-PI. **g** PC32:1. **h** PC34:1. **i** PC34:2. **j** PC36:3. **k** PC40:7. **l** PU-PC. * *P* < 0.05, ** *P* < 0.01

Total levels of phosphatidylcholine (PC) and phosphatidylethanolamine (PE), and the phospholipids phosphatidylserine (PS) and phosphatidylinositol (PI) were not changed in the tumors (Fig. 5a, b and Supplementary Fig. 1d). The PC/PE ratio was similar in tumor and non-tumor tissues (Fig. 5c).

Saturated, MU- and PU-PC were not changed in the tumors (Supplementary Fig. 1e). This also applied to MU-PE (data not shown) and PU-PE (Fig. 5d). Saturated PE and PS levels are very low and were not further analysed. MU-PS declined, and MU-PI was higher in the HCC tissues compared to the normal tissues whereas saturated PI, PU-PI and PU-PS levels did not change (Fig. 5e, f and data not shown).

There is some evidence that single PC species are changed in tumors [61]. The concentrations of the two MU-PC species (PC32:1, PC34:1) were similar in the non-tumor and tumor tissues (Fig. 5g, h). Regarding PU-species, PC34:2, 36:3 and 40:7 declined in the tumors whereas PC38:6 did not change (Fig. 5i-k and data not shown).Total PU-PC levels were similar in normal tissues and cancer tissues (Fig. 5l).

### Lysophosphospholipids and PE-plasmalogens are hardly changed in the tumors

**Fig. 6:**
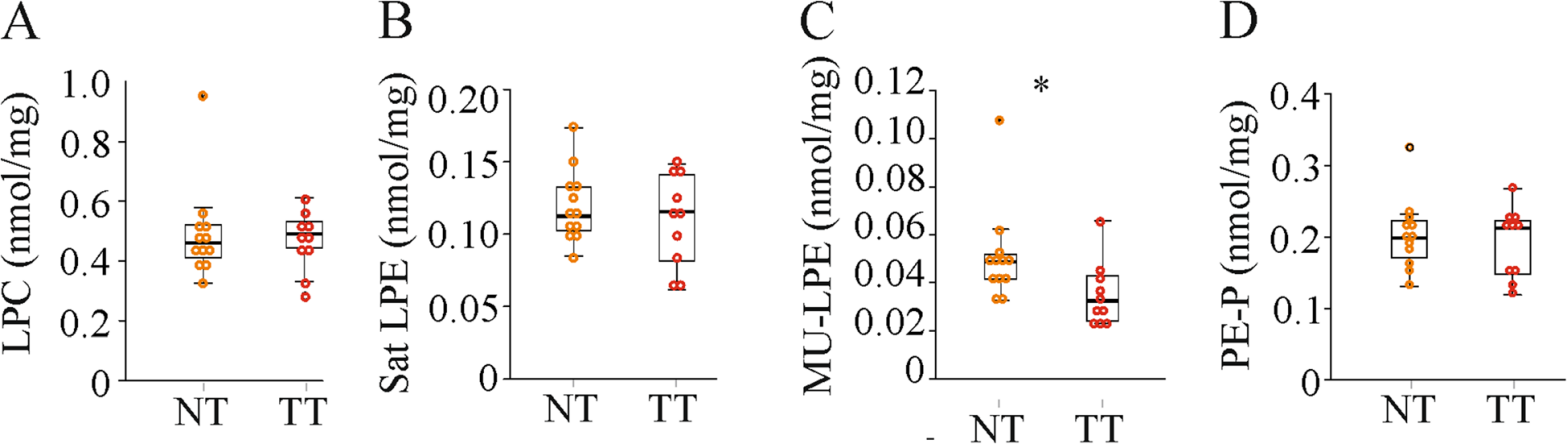
Hepatic lysophosphatidylcholine (LPC), lysophosphatidylethanolamine (LPE) and PE-plasmalogen (PE-P) levels in the normal tissues (NT) and tumor tissues (TT) of mice injected with diethylnitrosamine. (*n* = 10–12 mice). **a** LPC. **b** Saturated (Sat) LPE. **d** Monounsaturated (MU) LPE. **d** PE-P. * *P* < 0.05

Lysophosphatidylcholine (LPC) levels were not changed in the tumors (Fig. 6a). In terms of lysophosphatidylethanolamine (LPE) levels, saturated LPE was unaltered while MU-LPE levels declined in the tumor tissues (Fig. 6b, c). Besides, PE-plasmalogen levels were similar in tumor and non-tumor tissues (Fig. 6d).

### Correlation of PU-TG species with tumor number

Total TG, DG and CE concentrations were induced in the tumors (Figs. 1a and e and 3a). These lipid classes did not correlate with the number of tumors in the liver (Table 3). MU-PS, MU-PI and MU-LPE levels were also changed in the tumors (Figs. 5e and f and 6c). Again, these lipids did not correlate with tumor number. This also applied to ceramide and SM levels (Table 3).

**Table 3:** Spearman correlation coefficients and *P*-values for the correlation of the number of tumors per liver and the lipid classes, which were shown to be altered in tumor tissues in this study

Lipid class	r	*P*
TG	0.626	0.053
TG DB6	0.809	0.005
TG DB8	0.809	0.005
DG	0.517	0.126
CE	-0.036	0.939
Ceramide	0.116	0.751
SM	0.152	0.675
MU-PS	0.511	0.132
MU-PI	-0.109	0.763
MU-LPE	0.474	0.166

The nearly significant correlation of TGs with tumor number (Table 3) prompted more detailed analysis. There were indeed significant positive correlations of the PU-TG species with 6 and 8 double bonds and tumor number (Table 3).

### The tumor suppressors p53 is induced and the antioxidant enzyme MnSOD is reduced in the tumor tissues

**Fig. 7:**
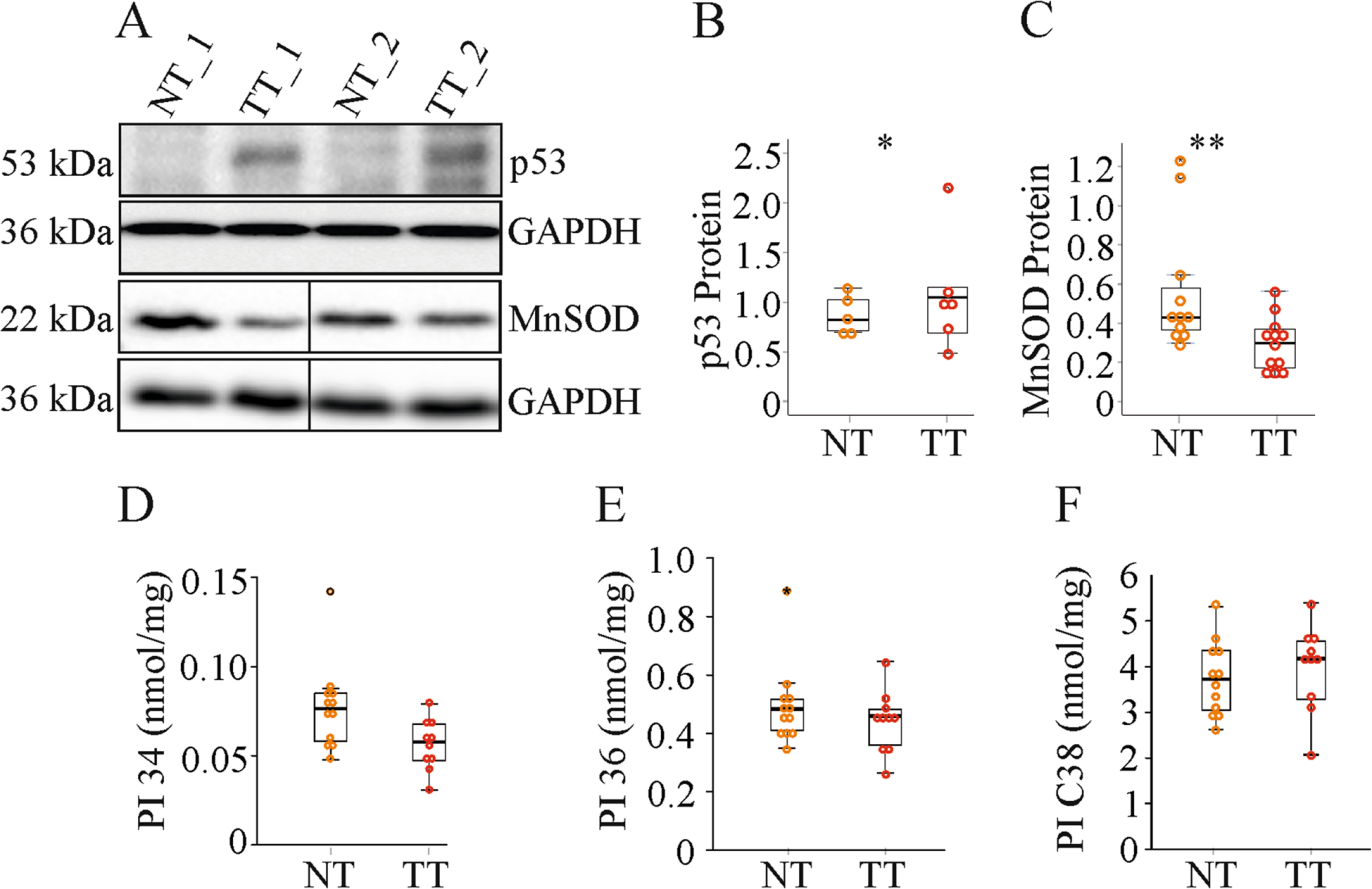
Hepatic expression of p53 and MnSOD and hepatic PI species related to p53 mutations in the normal tissues (NT) and tumor tissues (TT) of mice injected with diethylnitrosamine. **a** Expression of p53 and MnSOD. **b** Quantification of p53 (*n* = 5–6). **c** Quantification of MnSOD (*n* = 11–12). **d** PIs with 34 carbon atoms (*n* = 10–12). **e** PIs with 36 carbon atoms (*n* = 10 − 12). **f** PIs with 38 carbon atoms (*n* = 10–12). * *P* < 0.05, ** *P* < 0.01

The tumor suppressor protein p53 was increased in the murine tumors (Fig. 7a, b). One of the downstream targets of p53 is manganese superoxide dismutase (MnSOD) [62]. MnSOD protein was low in the HCC tissues (Fig. 7a, c). Mutant p53 causes a shift from C38 to C36 and C34 PI species [63]. This was not observed in the murine tumors (Fig. 7d - f).

## Discussion

Lipids play a fundamental role in the development and progression of HCCs [5, 7, 26]. DEN is a widely used chemical to induce HCC in rodents [40], and here, lipid profiling was performed on normal and cancer tissues. As shown in humans [5], TGs and DGs accumulated in the murine liver tumors. In contrast to human HCCs [7] key enzymes of *de novo* lipogenesis were not induced in the murine liver cancers. Of note, ceramides were increased in the murine tumors while a decline was noticed in human HCC tissues [5, 33]. Thus, the DEN-HCC model is not appropriate for testing novel drugs targeting *de novo* lipogenesis or ceramide metabolism [7, 33].

The main responsible transcription factor for *de novo* lipogenesis is SREBP1c, and FAS, ACC and SCD1 were induced in human liver cancer [4, 6, 64]. In contrast, an upregulation of these enzymes was not observed in the murine tumors. Likewise, DGAT1 and DGAT2 are frequently overexpressed in many cancers [65] while being unchanged in the murine liver tumors.

ACC is inactivated upon phosphorylation by AMPK, which induces catabolic pathways while blocking energy-consuming processes. AMPK is activated by AMP and by phosphorylation [66] and is supposed to exert tumor suppressive functions in HCC [67]. Levels of phosphorylated ACC and AMPK did not change in the tumors arguing against a critical role of these enzymes for the tumor-specific lipidome.

LPL is a further enzyme described to be high in human HCCs [9]. LPL enhances the uptake of fatty acids by tumor cells, and suppression of LPL reinforces the effect of FAS blockage on cell proliferation [9]. High expression of the fatty acid translocase CD36 in human HCC further supports a role for exogenous fatty acid uptake in HCC [68]. In fact, LPL and CD36 were even downregulated in the murine tumors. This largely excludes exogenous fatty acids as a source of cellular triglyceride accumulation in the tumors.

A decline of PGC1alpha was noted in the HCC tissues of the DEN injected mice. PGC1alpha increases mitochondrial biogenesis and enhances fatty acid oxidation [13, 21]. Low PGC1alpha in the murine tumors may contribute to lower TG deposition in the tissues. Besides, CPT2 mRNA levels were found reduced in the tumors. Expression of additional genes participating in fatty acid oxidation was not downregulated in the tumors. To prove the suggestion that fatty acid oxidation is impaired in the murine liver cancer tissues, functional analysis of fatty acid oxidation is required, which was not performed in the present study.

Notably, PGC1alpha suppressed ApoB expression in hepatocytes [13], and high ApoB protein in the rodent tumors may be related to low expression of PGC1alpha. PGC1alpha further enhances hepatocyte nuclear factor 4alpha-dependent activation of the LIPC promoter [69].

Deficiency of LIPC contributes to liver steatosis [70] and LIPC was indeed reduced in the murine tumors. Protein levels of a further lipase – HSL – were, however, not changed in the tumors.

PGC1alpha mRNA expression did not decline in parallel with protein levels proposing the involvement of post-transcriptional mechanisms. The tumor suppressor protein p53 decreases the stability of PGC1alpha [71], and high expression of p53 in HCC tissues may contribute to downregulation of PGC1alpha protein.

The carcinogen DEN is well described to upregulate p53 protein in the liver [72, 73]. Much less is known about an additional induction of p53 in the rodent HCC tissues. In mice and rats, p53 RNA was about 1.5 to 2-fold higher in the tumor tissues [74, 75]. The p53 protein was also increased in the HCC tissues of mice with non-alcoholic stesatohepatitis [43]. Accordingly, p53 protein was higher in the tumors of the mice studied herein.

One of the multiple functions of p53 is the regulation of MnSOD activity, an important mitochondrial antioxidant [62]. MnSOD was downregulated in the murine liver tumors and this was described in human HCC [76]. MnSOD is additionally regulated by PGC1 alpha, which is a potent inducer of this enzyme [16].

Strongly increased *de novo* lipogenesis in human HCCs causes an enrichment of saturated and MU-lipids at the expense of PU-lipids [24, 25]. Such a shift was not observed in the DEN-model. There was a modest change of relatively low abundant MU-lipids, and MU-PS decreased whereas MU-PI increased in the tumors. Hall et al. reported on a decline of PU-PC species in the murine tumors of DEN-injected mice [61], and three of the 12 examined PU-PC species were reduced in the tumors analysed herein. The observed upregulation of MU-PCs in murine and human HCCs [61] could not be confirmed in the present investigation. This recent study used C57BL/6 mice [61] whereas C3H/HeNRj mice were analyzed in the current investigation. Different mouse strains exhibit variations in their liver lipidome and in their response to diets [77]. In addition, the lipidome can be affected by age, gender, and circadian rhythm [78]. Comparative studies are needed to identify the links between the tumor lipidome, genetic and environmental traits.

Positive correlations between the degree of TG saturation and disease severity existed in human HCCs [25]. In contrast, a positive relationship of tumor number and PU-TG levels was noticed in the murine model. This suggests that PU rather than saturated or MU-TGs contribute to tumor growth in the mouse model.

The regulation of phospholipids in HCC tissues has not been finally clarified. It is likely that PU-species are lower in the tumors, and a decline of PC and PE levels was also described [79–81]. Of note, PE-plasmalogens were low in HCC tissues of patients and this may contribute to oxidative stress [5, 80, 82]. In the DEN model, none of the analysed phospholipid classes were largely changed in the tumors. This applied to PC, PE, PS, PI, LPE, LPC and PE-plasmalogen lipids. Mutant p53 was shown to affect PI acyl chain composition and to increase species with shorter-chain fatty acids [63]. A shift in PI species fatty acid length did neither occur in human HCC tissues [24] nor in the murine tumors.

Cholesterol enhances cell proliferation, and was induced in human HCC tissues [5]. Similar to the DEN model, CEs accumulated in the human tumor tissues whereas free cholesterol levels did not change [24]. The pathways contributing to cholesterol deposition in human HCCs differ between the patients. SREBP2 is the main transcription factor regulating cholesterol homeostasis and was activated in the tumors of some patients [5]. SREBP2 and the apolipoproteins E and AII, which both have a role in cholesterol efflux [83], were not changed in the murine cancer tissues. Here, as has been also described in human HCCs [32], LDL-receptor protein was increased. In the murine liver, LDL-R mRNA was not regulated in parallel indicating the involvement of post-transcriptional mechanisms. PCSK9 induces the degradation of the LDL-receptor and low PCSK9 expression in human HCCs is in agreement with higher LDL-receptor protein [32]. In contrast to human HCCs, PCSK9 protein was not suppressed in the murine tumors. The underlying pathways contributing to increased LDL-receptor protein in the HCC tissues despite an accumulation of CEs have still to be defined. Thus, higher expression of the LDL-receptor may contribute to cholesterol accumulation in murine and human tumors.

Long-chain ceramides induce cell death and very long-chain species have the opposite effect [5, 34]. Unexpectedly, most of the ceramide species were induced in the murine tumors, and the long-chain / very long-chain ceramide ratio was increased. The decline of SMs in the murine tumors reveals an involvement of sphingomyelinases [5, 84]. In strong contrast to the murine situation, in most human HCC tissues ceramide levels are low while SM concentrations increase [5, 26, 85]. Thus the animal model described herein may be used to study the molecular adaption of liver tumor cells, which succeed to survive and proliferate despite high endogenous ceramide levels.

HCCs are heterogeneous tumors and disease etiology may affect tumor-associated lipid composition [85]. DEN-injected C3H/HeNRj mice fed a low methionine, choline-deficient diet were used to study HCC development in non-alcoholic steatohepatitis. Notably, *de novo* lipogenesis was suppressed in these tumors and levels of TGs and DGs were low [43]. Various murine HCC models were established [40] and lipidomic profiling will give further insights into the role of dysregulated lipid metabolism in cancer development and progression.

### Study strengths and limitations

The strength of this study was the comprehensive analysis of the cancer associated lipid composition in the widely used DEN model. Expression of several proteins with a role in lipid metabolism was analysed in parallel. Limitation is that only male C3H/HeNRj mice fed a standard chow were studied. Comparison of mouse strains or mice fed different diets was not performed. Besides, fatty acid synthesis, oxidation and uptake by the tumors were not quantified.

## Conclusions

The DEN injected mice had higher TG levels in the tumors as was reported in human HCCs. *De novo* lipogenesis did not increase in parallel, and a shift from PU to saturated lipids was not observed. Thus, anti-HCC drugs targeting FAS or ACC may not be effective in this model. Ceramides did not decline in the tumors, but were rather induced. The model described herein is thus suitable to identify the molecular changes allowing hepatocyte proliferation when cellular ceramides are increased. This may be relevant for the development of anticancer drugs targeting the sphingolipid pathways.

## Supplementary Information

**Additional file 1: Supplementary Figure 1.** Expression of SREBPs and SCD1 and median values of CEs and phospholipids in the normal tissues (NT) and tumor tissues (TT) of mice injected with diethylnitrosamine. **a** Expression of SREBP1c precursor and active form and SCD1. **b** Hepatic cholesteryl ester (CE) species and free cholesterol (FC). **c** Expression of SREBP2 precursor and active form. **d** Median levels of PS and PI in TT and NT. **e** Median levels of saturated (sat), MU-PC and PU-PC in NT and TT. ** *P* < 0.01.

## Data Availability

The datasets generated and/or analyzed during the current study are available from the corresponding author on request.

## Abbreviations

ACC: Acetyl-CoA-carboxylase
AMPK: AMP-activated protein kinase
Apo: Apolipoprotein
CE: Cholesteryl ester
DG: Diglycerides
DGAT: Diacylglycerol-O-acyltransferase
FAS: Fatty Acid Synthase
HSL: Hormone sensitive lipase
LIPC: Hepatic lipase
LPL: Lipoprotein Lipase
LDL: Low density lipoprotein
LPC: Lysophosphatidylcholine
LPE: Lysophosphatidylethanolamine
MnSOD: Manganese Superoxide Dismutase
PC: Phosphatidylcholine
PCSK9: Proprotein convertase subtilisin/kexin type 9
PE-P: PE-plasmalogen
PE: Phosphatidylethanolamine
PGC1alpha: Peroxisome proliferator-activated receptor gamma coactivator 1 alpha
PI: Phosphatidylinositol
PS: Phosphatidylserine
SM: Sphingomyelin
TG: Triglycerides
