# Supplementary figures and images for: Accumulation of cholesterol, triglycerides and ceramides in hepatocellular carcinomas of diethylnitrosamine injected mice
Source: Lipids Health Dis. 2021 Oct 10;20:135. doi: 10.1186/s12944-021-01567-w (PMC8502393; doi:10.1186/s12944-021-01567-w)

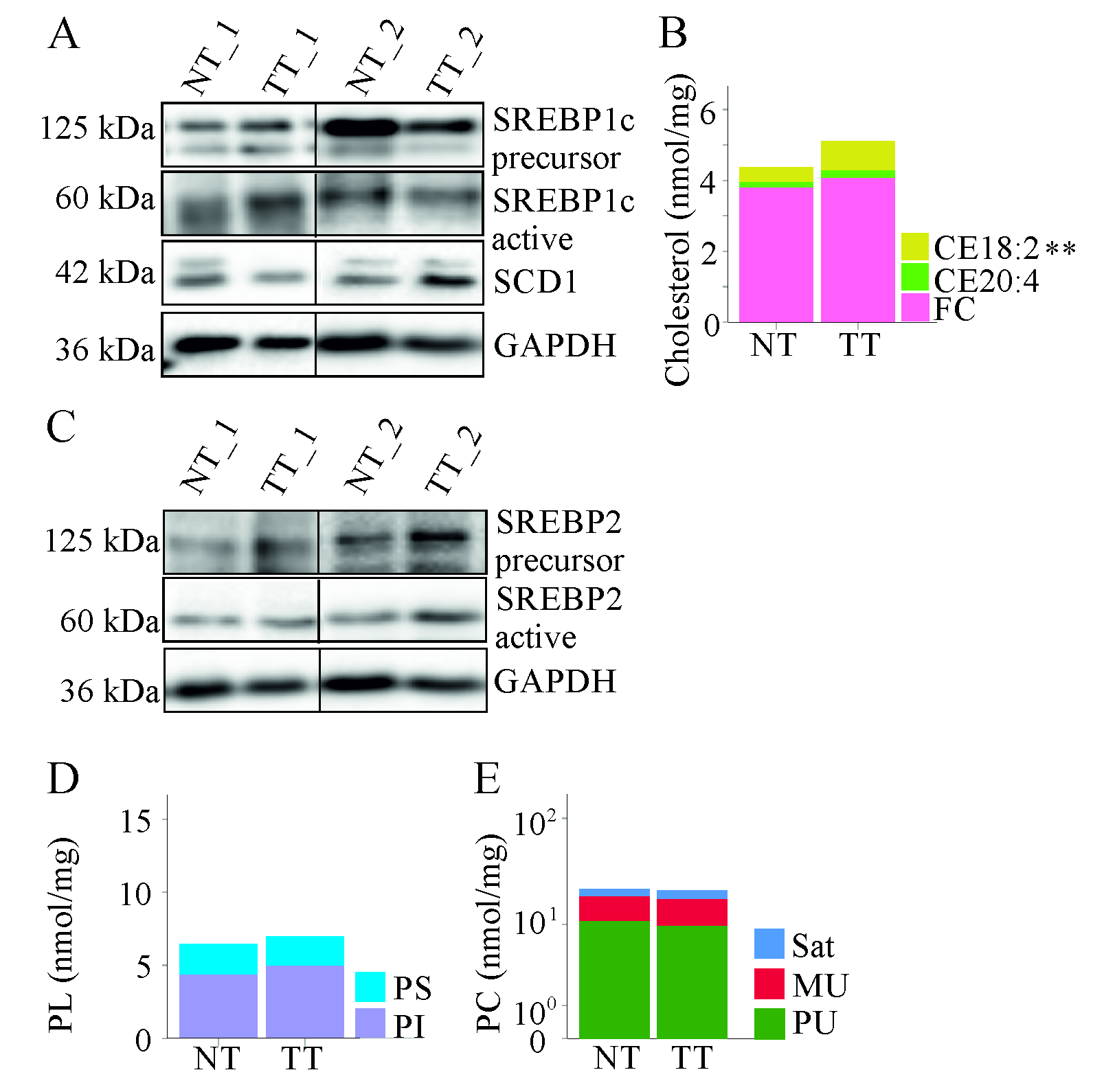

Supplement: Supplementary file 1 — Additional file 1: Supplementary Figure 1. Expression of SREBPs and SCD1 and median values of CEs and phospholipids in the normal tissues (NT) and tumor tissues (TT) of mice injected with diethylnitrosamine. a Expression of SREBP1c precursor and active form and SCD1. b Hepatic cholesteryl ester (CE) species and free cholesterol (FC). c Expression of SREBP2 precursor and active form. d Median levels of PS and PI in TT and NT. e Median levels of saturated (sat), MU-PC and PU-PC in NT and TT. ** P < 0.01. [file 12944_2021_1567_MOESM1_ESM.tif]
